# Aggregating single nucleotide polymorphisms improves filtering for false-positive associations postimputation

**DOI:** 10.1093/g3journal/jkaf043

**Published:** 2025-03-07

**Authors:** Katharina Stahl, Sergi Papiol, Monika Budde, Maria Heilbronner, Mojtaba Oraki Kohshour, Peter Falkai, Thomas G Schulze, Urs Heilbronner, Heike Bickeböller

**Affiliations:** Department of Genetic Epidemiology, University Medical Center Göttingen, Göttingen 37073, Germany; Institute of Psychiatric Phenomics and Genomics (IPPG), LMU University Hospital, Ludwig Maximilian University of Munich, Munich 80336, Germany; Department of Psychiatry and Psychotherapy, LMU University Hospital, Ludwig Maximilian University of Munich, Munich 80336, Germany; Department Clinical Translation, Max Planck Institute of Psychiatry, Munich 80804, Germany; Institute of Psychiatric Phenomics and Genomics (IPPG), LMU University Hospital, Ludwig Maximilian University of Munich, Munich 80336, Germany; Institute of Psychiatric Phenomics and Genomics (IPPG), LMU University Hospital, Ludwig Maximilian University of Munich, Munich 80336, Germany; Institute of Psychiatric Phenomics and Genomics (IPPG), LMU University Hospital, Ludwig Maximilian University of Munich, Munich 80336, Germany; Department Clinical Translation, Max Planck Institute of Psychiatry, Munich 80804, Germany; Department of Immunology, Faculty of Medicine, Ahvaz Jundishapur University of Medical Sciences, Ahvaz 61357-15794, Iran; Department of Psychiatry and Psychotherapy, LMU University Hospital, Ludwig Maximilian University of Munich, Munich 80336, Germany; Department Clinical Translation, Max Planck Institute of Psychiatry, Munich 80804, Germany; German Center for Mental Health (DZPG), partner site Munich/Augsburg, Munich 80336, Germany; Institute of Psychiatric Phenomics and Genomics (IPPG), LMU University Hospital, Ludwig Maximilian University of Munich, Munich 80336, Germany; German Center for Mental Health (DZPG), partner site Munich/Augsburg, Munich 80336, Germany; Department of Psychiatry and Behavioral Sciences, SUNY Upstate Medical University, Syracuse, NY 13210, USA; Department of Psychiatry and Behavioral Sciences, Johns Hopkins University School of Medicine, Johns Hopkins University, Baltimore, MD 21287, USA; Institute of Psychiatric Phenomics and Genomics (IPPG), LMU University Hospital, Ludwig Maximilian University of Munich, Munich 80336, Germany; Department of Genetic Epidemiology, University Medical Center Göttingen, Göttingen 37073, Germany

**Keywords:** genotype imputation, genome-wide association, simulation study, false-positive results, quality control

## Abstract

Imputation causes bias in *P*-values in downstream genome-wide association studies. Imputation quality measures such as IMPUTE info are used to discriminate between false and true associations. However, implementing a high threshold often discards true associations, while a low threshold preserves false associations. This poses a challenge, especially for studies genotyped with SNP arrays. In practice, association signals register as spikes of low *P*-values for SNPs in close proximity owing to linkage disequilibrium, but postimputation filtering is conducted on SNPs independently. We simulated 1536 small case–control studies on the human chromosome 19 both to quantify the introduced bias and to evaluate postimputation filtering. The established IMPUTE info thresholds 0.3 and 0.8 were compared on individual SNPs and aggregated spikes in the formats “best guess genotype” and “dosage.” Furthermore, we applied 2 recently published methods, Iam hiQ and MagicalRsq, to assess their effect on filtering. We found differences in false signals and imputation quality between the genotype formats, especially in the midrange between thresholds. In this midrange, 51 and 60% of associated SNPs for best guess and dosage format, respectively, are true associations. For aggregated SNPs, the majority of spikes in the midrange are true associations. We propose a new method, the Midrange Filter, which uses both thresholds and formats to classify spikes instead of SNPs. This method discards up to the same number of false signals as the upper threshold, while preserving all true associations in most simulation settings. The PsyCourse study is included as a real-data application.

## Introduction

Genotype imputation is a well-established practice to increase power in genome-wide association studies (GWASs), enable meta-analysis in studies genotyped on different SNP arrays, and discover entirely untyped signals (Li *et al*. 2008, 2009; Marchini and Howie 2010; Pei *et al*. 2010). Several imputation algorithms utilize fully sequenced reference panels to infer the missing SNPs in the target datasets (Howie *et al*. 2009, 2012; Das *et al*. 2016; Browning *et al*. 2018; Bycroft *et al*. 2018). Even though overall imputation accuracy is high, an inherent uncertainty in the estimation remains (Shi *et al*. 2018; Stahl *et al*. 2021). Most methods of analysis based on genotypes do not allow the direct use of probabilities representing the possible genotypes of each SNP, which is why they are usually collapsed into the expected count of alternative alleles (dosage) or the genotype with the highest genotype probability (best guess). Both genotype formats lose information concerning the uncertainty of imputation, although this loss is more profound in best guess than in dosage.

The confidence in the imputation is expressed by imputation quality measures based on the probabilities of each possible genotype per SNP, e.g. IMPUTE info (Marchini and Howie 2010). However, using only SNPs with a high imputation quality does not guarantee unbiased downstream analysis, since the true accuracy of imputation is impossible to determine without knowing the true underlying genotypes (Roshyara *et al*. 2014; Stahl *et al*. 2021). For SNPs with a low minor allele frequency (MAF), missed rare variants are not well reflected in imputation quality measures (Shi *et al*. 2018; Stahl *et al*. 2021), but impact association results. Furthermore, imputation quality measures cannot discriminate false-positive and true-positive associations perfectly (Zhang *et al*. 2022). A lower threshold favors the preservation of true-positive associations, while a high and therefore more stringent threshold favors the elimination of false-positive associations. As a result, there is no universal recommendation for imputation quality thresholds. For example, both 0.3 (Li *et al*. 2010) and 0.8 (Nelson *et al*. 2013) are established thresholds for IMPUTE info, although a variety of thresholds are used in practice to accommodate study settings and goals (Naj 2019). Since imputation quality has improved with new versions of imputation programs, stringent thresholds have become more common threshold in recent studies but is still not implemented universally (Naj 2019).

The issue of sacrificing true associations in order to eliminate false-positive findings in downstream analysis was recently quantified, finding a threshold of 0.8 for Beagle R² to discard a large number of true-positive SNPs if the proportion of imputed SNPs is high (Zhang *et al*. 2022). While the simulation settings of the study (Zhang *et al*. 2022) are comparable with complementing SNP arrays for joint meta-analysis, the deletion pattern is not reflective for imputing SNPs from a genotyping array for GWAS. Imputation based on a SNP array has a smaller scaffold of genotyped SNPs, which impacts imputation quality (Roshyara *et al*. 2014), and the placement of known SNPs is not random. Small studies with a low number of participants and a high number of imputed SNPs might be impacted even more (Roshyara *et al*. 2014).

The consequences of discarding or preserving significant SNPs in a GWAS setting depend on the number of discarded SNPs and their relation. Owing to linkage disequilibrium (LD), a true signal in a sufficiently densely genotyped GWAS does not consist of one singular SNP that passes the significance threshold, but a group of SNPs in close physical distance producing a spike of low *P*-values in association tests, which are visible in Manhattan plots. Effective filtering in this context discards whole spikes comprising entirely false-positive SNPs and preserves spikes containing true-positive SNPs as much as possible. Imputation itself is carried out with a sliding window to capture LD better, but filtering for imputation quality is carried out on individual (Naj 2019; Truong *et al*. 2022).

We designed a simulation study based on the 1000 Genomes Project (1KGP) (Auton *et al*. 2015) to investigate the effect of imputation on downstream GWAS and to improve filtering of false-positive associations by aggregating SNPs into spikes. Contrasting previous research (Zhang *et al*. 2022), we used existing SNP arrays as basis for imputation. The association was carried out on best guess genotypes and dosage to investigate the effect of preserved uncertainty. We quantified the bias of *P*-values and resulting false-positive associations, comparing the results of filtering with imputation quality measures on individual SNPs and on spikes. With the use of spikes, we derived a filtering method to refine filtering for association results, which was named the Midrange Filter. The Midrange Filter leverages LD and differences between genotype format to identify spikes in the midrange of imputation quality, i.e. an IMPUTE info score between 0.3 and 0.8, as true- or false-positive association signals. We also tested 2 recently published methods to improve filtering SNPs with imputation quality measures, namely Iam hiQ (Rosenberger *et al*. 2022) and MagicalRsq (Sun, Yang, *et al.* 2022). We conducted a GWAS on the PsyCourse study as a real-data application for the imputation quality control methods (Budde *et al*. 2017).

## Materials and methods

### Data and simulation

All computations were performed on the High Performance Computing cluster of the Gesellschaft für wissenschaftliche Datenverarbeitung mbH Göttingen with R (R Core Team 2022) as a framework and the R package batchtools (Bischl *et al*. 2015; Lang *et al*. 2017) for parallelization. Bcftools was used for data handling, annotation, and formatting (Danecek *et al*. 2021).

The simulations are a variety of case–control studies, which were created with HapGen2 (Su *et al*. 2011) and based on the 1KGP Phase 3 dataset (Auton *et al*. 2015) limited to chromosome 19 for scope. Chromosome 19 contains 638,810 SNPs for 2,504 samples.

The original 2,504 samples were divided randomly into even halves to create different pools of individuals for the simulation of target dataset and the reference panel. To generate imputed SNPs, a portion of the target dataset was deleted, while a copy of the whole simulated data was kept intact for later comparison.

Imputation and phasing were carried out with Beagle5.2 for its accuracy and speed (Browning *et al*. 2018; Stahl *et al*. 2021). Both the initially simulated and the imputed datasets were tested for association with a logistic regression model implemented in R. The significance level is set to 5e−8, as is common for GWAS (Fadista *et al*. 2016). The association was performed on both on dosage and best guess.

The complete simulated dataset and the resulting *P*-values after association are treated as the gold standard for each iteration. For clarity and brevity, those results are referred to as “true” in comparison with the imputed results.

The simulation settings are varied by adjusting several input variables. By design, these variations include scenarios, which may influence imputation quality. In this way, we aim to produce both true and false signals as close to a real-data example as possible to be able to effectively evaluate the imputation quality methods.

#### Position and effect size of disease loci

Four sets of disease loci and effect sizes are introduced. For each set, the disease loci were picked from randomly selected SNPs to control physical position and MAF. While the MAFs of the disease loci are varying due to the random picking, we only used the range between 0.05 and 0.4 in the original dataset to ensure a level of discoverability of true significant SNPs even for smaller effect sizes. Sets 1 and 2 contain SNPs spread evenly across chromosome 19, varying between odds ratios of 1.5 and 3 and dominance models. Set 3 consists of disease loci located at the physical edge of the chromosome and contains smaller effects, which interferes with imputation quality and therefore power to find true associations (Stahl *et al*. 2021). Each simulation in sets 1, 2, and 3 uses 1 disease locus paired with each effect size. Set 4 uses the same disease loci and effect sizes as sets 1 and 2, but contains 2 or 3 disease loci instead of 1 for every simulation. These variations were included to ensure our results were not dependent on picking one specific disease locus.

#### Deletion pattern

There are 3 settings for the deletion of SNPs based on SNP arrays. The SNP arrays Illumina Infinium Omni5-4 kit, the Illumina Infinium Omni2.5-8 v1.5, and the Infinium OmniExpress-24 Kit were used to vary the density of the scaffold (Ha *et al*. 2014). The arrays contain 90,172, 48,597, and 14,729 SNPs on chromosome 19, respectively. More specific information about the arrays may be found at the Illumina website (www.illumina.com). Following the parameters of previous research (Zhang *et al*. 2022), we also simulated randomly deleting 80% of SNPs with a uniform distribution, the results of which are presented in the section “Random Deletion” in the supplementary material.

#### Number of participants

The initial setting simulates 1,000 individuals in each study group, which is inspired by common clinical practice. Reference panels comprising 10,000 individuals are simulated separately for each group and iteration. As rare disease cases are harder to recruit than controls, scenarios limited to 333 individuals for the case group were included. Furthermore, simulations with reference panels consisting of 5,000 instead of 10,000 simulated individuals were also included.

#### Genetic distance

The majority of simulations divided the 1KGP data into the basis for test and reference data, so that no subpopulation is sparse in either half. To simulate a greater genetic distance between references and controls, we included simulations, where one subpopulation is strictly used only for simulating either the reference or the test dataset. This way, there are individuals contained in the reference with the same superpopulation as the test dataset, but not of the specific subpopulation. The reference panels in those settings are somewhat mismatched, but not to an unrealistic degree.

In total, 1,536 case–control settings were simulated with SNP arrays as the basis of imputation. Details on the settings are listed in Supplementary Tables 5–7 for reproducibility.

### Imputation quality and accuracy for individual SNPs

The simulation allows comparison of the accuracy of filtering GWAS results with postimputation quality measures and their proposed thresholds.

The most common imputation quality measures are IMPUTE info score, Beagle R^2^, and MaCH R^2^ to estimate imputation quality without access to the underlying genotypes (Browning and Browning 2009; Marchini and Howie 2010; Das *et al*. 2018). These 3 quality measures are highly correlated (Marchini and Howie 2010; Stahl *et al*. 2021). In previous research, IMPUTE info recognized differences in imputation quality slightly better and was therefore used to present the results (Stahl *et al*. 2021). The IMPUTE info score estimates the ratio between observed and expected statistical information. Similar to the score test (Rao 2005), it estimates the expected Fisher information and variance of the U score with the distribution of imputed genotypes for each SNP. IMPUTE info is low if the mean estimated variance of the imputed genotypes is close to the variance of genotypes sampled by chance with MAF (Marchini and Howie 2010). The effectiveness of filtering false- and true-positive associations with IMPUTE info thresholds of 0.3 and 0.8 was tested. These thresholds are also used to define the midrange of imputation quality.

The imputation quality measures were calculated separately for cases and controls, as the imputation itself is a case–control simulation. This results in 2 scores per SNP. In the subsequent assessment of imputation quality control methods, the minimum of the scores was used to represent the imputation quality for each SNP. All imputation quality measures except MagicalRsq were calculated with ImputAccur (Thormann *et al*. 2022).

Depending on the study setting, deleting SNPs with an MAF smaller than 0.05 is recommended (Truong *et al*. 2022). Similar to the imputation quality threshold, setting a cutoff for MAF that is not specific for the dataset is arbitrary and does not necessarily yield optimal results (Tabangin *et al*. 2009; Charon *et al*. 2021). Given the small sample size of our simulation, SNPs with low MAF were not excluded.

### Refining imputation quality with Iam hiQ and MagicalRsq

The recently published imputation quality measures Iam hiQ (Rosenberger *et al*. 2022) and the calibration method MagicalRsq (Sun, Yang, *et al.* 2022) were also assessed to improve quality control.

Iam hiQ is a novel pair of imputation accuracy measures with intuitive interpretation, which is recommended to be used in addition to the established measures such as IMPUTE info (Rosenberger *et al*. 2022).

Iam quantifies the proportion of individual-specific genotype information compared with the information from the reference population. There are 2 variants of Iam: Iam_chance_ and Iam_hwe_. An SNP receives a low Iam_chance_ score if the genotype probabilities per individual are mostly uniform. This indicates largely random imputation. Iam_hwe_ scores low if genotype probabilities were derived largely by allele frequency according to Hardy-Weinberg equilibrium (HWE). In both cases, the imputation is not specific to the individuals. The recommended threshold is 0.5 for both Iam score. The extreme cases of low Iam scores are mutually exclusive, and Iam_hwe_ cannot surpass Iam_chance_ (Rosenberger *et al*. 2022). In addition to the individual measures, we combined the 2 scores into 1:


$$\mathrm{Ia}m_{\mathrm{combined}}=1\text{–}(\mathrm{Ia}m_{\mathrm{chance}}\text{-}\;\mathrm{Ia}m_{\mathrm{hwe}})=\mathrm{Ia}m_{\mathrm{hwe}}\text{-}\;\mathrm{Ia}m_{\mathrm{chance}}+1$$

The combined score ranges from 0 to 1 with a high score indicating imputation based on individual-specific information. Since Iam scores have not been combined in this way, to our knowledge, there is no recommended threshold.

HiQ describes the heterogeneity of genotype probabilities between individuals. An SNP scores low if this heterogeneity is lacking owing to, e.g. rare variants leading to largely monomorphic imputation. Such SNPs are uninformative in further analysis such as GWAS. For quality filtering, a threshold of 0.9 is recommended (Rosenberger *et al*. 2022).

MagicalRsq is a calibration of imputation quality measures to improve accuracy with machine learning methods (Sun, Yang, *et al.* 2022). Pretrained models and additional population-based information are leveraged to better reflect the unknown true *R*^2^. For effective use, the models are trained on a subset of fully sequenced individuals within a dataset or on additional directly genotyped SNPs in all individuals, so the calibration can gauge the difference between the imputed R² and the real R² (Sun, Yang, *et al.* 2022). This work is focused on small clinical settings, where such additional information is likely unavailable. There are 4 models available with the implementation of MagicalRsq, which were used instead. The models were trained on a subset of UK Biobank with African descent (Bycroft *et al*. 2018), Cystic Fibrosis Genome Project (Sun, Liu, *et al.* 2022), the 1KGP (Auton *et al*. 2015), and the TOPMed dataset (Taliun *et al*. 2021). The 1KGP and the TOPMed data were used as a reference panel for training the models (Sun, Yang, *et al.* 2022). A number of SNPs were removed in the processing of MagicalRsq because no information about population-specific allele frequencies could be added for them. Therefore, results of calibrated imputation quality with and without this information are included. As in the original publication (Sun, Yang, *et al.* 2022), MaCH R² was adjusted. We used the published models to test the performance of MagicalRsq for studies where the model cannot be trained on the study dataset.

### Aggregation of SNPs to spikes

**Fig. 1.**
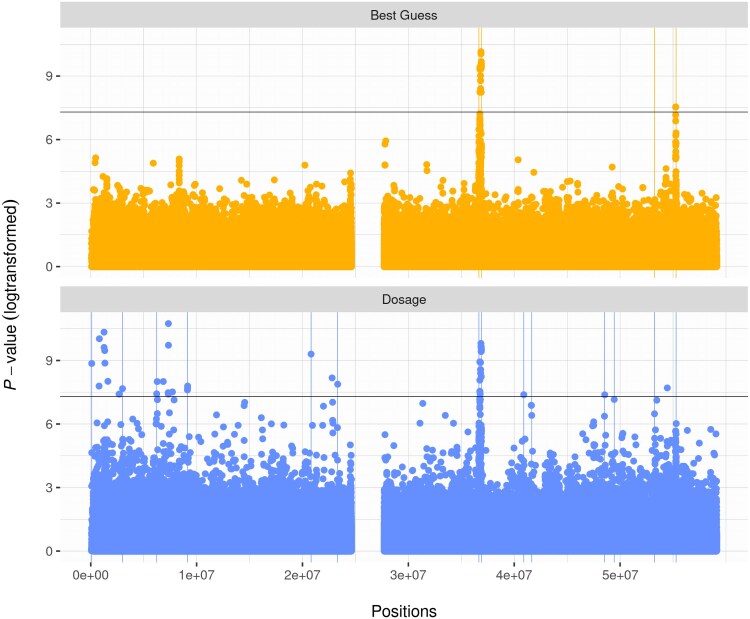
Manhattan plot of example data in best guess and dosage format (chromosome 19). The vertical lines represent the edges of the spikes determined with the aggregation algorithm described in the *Materials and Methods* section. Therefore, all SNPs between the vertical lines are assigned to the same spike. The horizontal line represents the significance threshold of 5e−8.

A simple algorithm was implemented to aggregate significant SNPs to spikes by physical proximity. The algorithm uses solely a list of positions without regarding the *P*-value. SNPs are assigned to the same spike, if they are fewer than 2 Mb apart or if they are connected by a chain of SNPs with each SNP fewer than 2 Mb apart from its neighbor. To determine the number and location of spikes more accurately, the positions of SNPs with a *P*-value below 5e−7 were used in the algorithm instead of SNPs reaching the genome-wide significance threshold of 5e−8. This concurs with the intention to consider SNPs in close proximity in support of an association signal as a whole. The thresholds of 5e−7 and 2 Mb were derived empirically. The aggregation algorithm was run on both best guess genotype and dosage, and the resulting spikes were checked for their presence in either format. An implementation in R including a test dataset is available on Github (github.com/StahlKt/MidrangeFilter), the results of which are depicted in Fig. 1.

### Midrange Filter

The Midrange Filter is a new filtering method to identify false-positive spikes. It leverages LD and the loss of information between best guess and dosage.

The choice of genotype format has the potential to influence association results, leading to format-specific associations in some SNPs. Dosage has a greater power to discover associations than best guess, since best guess loses all uncertainty of the genotype probabilities. However, flattening the uncertainty of imputation into best guess potentially removes random noise and may enable the discovery of association signals. By definition, the midrange does not focus on SNPs with high confidence, so the difference between formats is more pronounced and can be leveraged. Given the LD between SNPs of a spike and the mutual influence of nearby SNPs during imputation, both the maximum and minimum of imputation quality of significant SNPs in a spike are informative for the confidence in the whole spike.

Two thresholds for imputation quality are set, 1 threshold as the minimum of acceptable imputation quality and 1 threshold for high-confidence imputation quality. Spikes including significant typed SNPs are regarded as true, because of their proximity of a secure significant SNP, and therefore kept. Spikes including only imputed SNPs are further assessed. First, spikes are classified into 2 types: either they are discoverable at least in the dosage format (case 1) or they are format specific to best guess (case 2). Any spike is discarded if the SNP with the lowest imputation quality does not meet the lower threshold. Because of the higher power of the dosage format, confidence in the imputation of a best guess–specific spike needs to be higher compared with a dosage spike. Therefore, a best guess–specific spike is discarded if the SNP with the highest imputation quality does not meet the higher threshold.

**Fig. 2.**
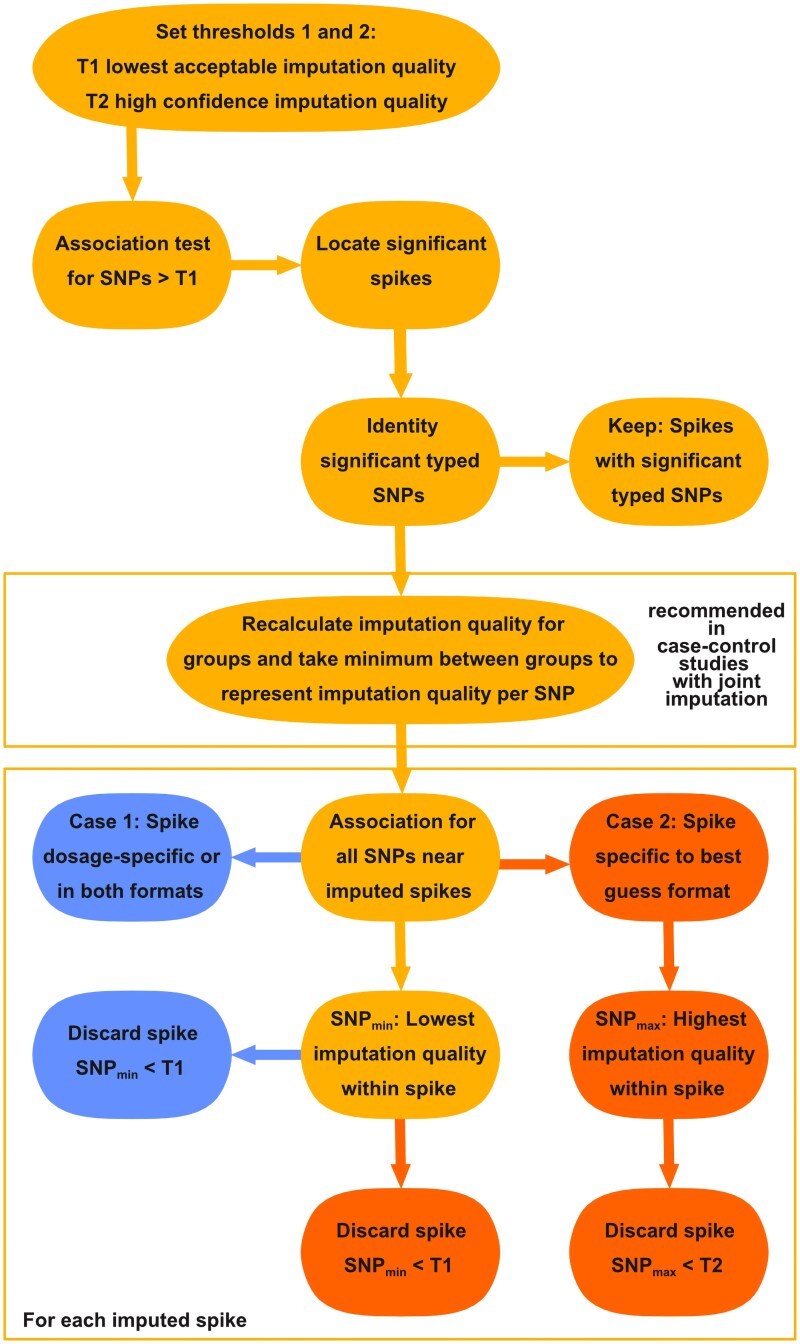
Flowchart of the Midrange Filter. After the additional association for SNPs surrounding imputed spikes, spikes are categorized into case 1 or 2 within the box labeled with "for all spikes". All steps pertaining case 1 are on the left side in the box. All Spikes falling into case 2 have an addditional steps outlined on the right side.

For practical use of the midrange filter, associations are carried out for SNPs above the minimum of acceptable imputation quality in the desired genotype format. If significant SNPs are found, they are validated by additional association testing on the SNPs surrounding those spikes. If the initial association test is based on dosage, the follow-up association is carried out in the dosage format only for SNPs below the initial threshold. If the initial association test is based on the best guess genotype, SNPs of all imputation quality are tested for the follow-up association in dosage, so that spikes may be classified for the midrange filter. If cases and controls are not imputed separately, imputation quality should be recalculated separately to determine if the imputation performed adequately for both groups. See Fig. 2 for an illustration of the workflow.

### Application of Midrange Filter—PsyCourse study

As a practical application, we used the Midrange Filter as a postimputation quality control method for association analysis in the PsyCourse study. The Psycourse study is a multicentric case–control longitudinal study based in Germany and Austria (Budde *et al*. 2017). Cases comprise of patients diagnosed with disorders on the affective-to-psychotic spectrum. PsyCourse is a deep-phenotyping study that assessed several phenotypes related to cognitive performance and symptom severity, which were tracked over the course of 4 visits about 6 months apart each (PsyCourse Codebook—Version 6.0). All participants gave written informed consent. For our application, we used version 6 of the PsyCourse dataset, which includes 591 genotyped cases diagnosed with bipolar disorder and depression (affective group), 530 cases diagnosed with disorders causing psychotic episodes such as schizophrenia (psychotic group), and 404 control individuals. The total number of participants (*n* = 1595) fits our simulation setting. The participants were genotyped with the Illumina Infinium Global Screening Array-24 Kit (versions 1 and 3). The genetic data used in the association are in hg19. For a simple GWAS model, we used the phenotypes at the baseline visit only. We modeled the case–control status with logistic regression, pooling the psychotic and affective group, as follows:


$$\begin{array}{rl} Logit(P(Y_{\mathrm{status}}=1)) & =\;\beta_{0}+\;\beta_{1}\;*\;\mathrm{SNP}+\;\beta_{2}\;*\;\mathrm{gender}\\ & \;+\;\beta_{3}\;*\;\mathrm{age}+\;\overset{5}{\underset{i=1}{\sum }}\;\beta_{i+3}\;*\;PC_{i}+\;\varepsilon \end{array}$$

Gender and age as they were assessed on the first visit were included for confounders. Further, we included the first 5 principal components to correct for population stratification. All SNPs were associated in dosage and best guess format for comparison.

We tested for association with a significance threshold of 5e−8. Cases and control were imputed jointly on the Helmholtz Munich Imputation Server, using EAGLE 2.4 for phasing and minimac4.1.6 for imputation with the Haplotype Reference Consortium data as a reference panel. To use the Midrange Filter as closely to our setting as possible, we recalculated imputation quality for the diagnostic groups (affective cases, psychotic cases, and controls) separately. We set the 2 thresholds for the midrange filter as 0.3 and 0.8, which are then contrasted to the provided imputation quality measure. To reduce the computational load and number of association tests, first only SNPs with an initial imputation quality above 0.3 were tested for association. If significant SNPs were found, SNPs with lower imputation quality within 2 Mbp of significant SNPs were tested for associations.

## Results

### Inflation and false-positive association

All deleted SNPs were recovered in the imputation, regardless of setting. A SNP and its association are considered false, if the level of significance is met only in the imputed dataset, but not in the underlying simulation. Given the strong differences between random deletion and SNP array simulations, imputation quality was evaluated only for SNP array simulations. The contrasting results between SNP array settings and random deletions are presented and discussed in Supplementary Table 1 and Fig. 1.

In general, the different simulation settings produced similar and expected results. False associations are more common in simulations where the reference panel is either smaller or does not match the subpopulations properly, although falsely associated SNPs are not exclusively appearing in these settings. As expected, simulations with a low number of cases, small effect sizes, or disease loci at the tail end of the chromosome suffer from a loss of power. Therefore, in 728 simulations, no SNPs were significant. We did not find any remarkable differences between the SNP arrays.

The bias of *P*-value does not appear as a 1-sided inflation for SNP array simulations. The imputed *P*-value was fairly close to the true *P*-value in the majority of simulations. In 240 simulation scenarios, some preimputation significant SNPs could not be identified as such after imputation and in 65 could not identify any previously significant SNP after imputation. The dosage format not only introduces more false positives but also identifies more true positives than best guess.

### Filtering with imputation quality on SNPs individually

Table 1 illustrates the results of the established thresholds with IMPUTE info. The number of significant SNPs are stated in both formats because of the presence of format-specific associations, i.e. the association is significant in best guess or dosage, but not both. Using the IMPUTE info for imputation quality, the midrange between thresholds contained 163 SNPs (107 and 145 in best guess format and dosage format, respectively), which amounts to about 1% of total significant SNPs. However, discarding these SNPs results in approximately equal proportions of right and wrong decisions or worse: about 49% (best guess) and 40% (dosage) of the SNPs in the midrange are false positives.

**Table 1.** Results of filtering the imputed significant SNPs with IMPUTE info at 0.3 and 0.8, separated by genotype format of the SNPs.

	Best guess SNPs				Dosage SNPs			
	Total	False	True	False %	Total	False	True	False %
All	14,208	979	13,229	06.89	14,796	1369	13,427	09.25
>0.8 IMPUTE info	14,101	927	13,174	06.57	14,362	1024	13,338	07.13
>0.3 IMPUTE info	14,208	979	13,229	06.89	14,509	1082	13,327	07.46
In midrange	107	52	55	48.60	145	58	87	40.00

The error column False % depicts the ratio between the false-positive and total SNPs in percentage.

For Beagle R^2^ and MaCH R^2^, 38% of SNPs at best in the midrange are false positive. All SNPs discarded by the lower threshold are format specific to dosage and false positive. Given both the low MAF and low imputation quality, these are easily identifiable by all quality control methods.

### Refining imputation quality with Iam hiQ and MagicalRsq

Iam may be used in combination with IMPUTE info either exclusively or inclusively: either SNPs have to pass the threshold for both IMPUTE info and Iam scores or SNPs only have to pass one to remain in the dataset. In the latter case, Iam functions as a failsafe for a potentially too high cutoff. Using Iam exclusively with the low IMPUTE info threshold and using Iam inclusively with the high IMPUTE info threshold yield the same result. Other combinations do not change the initial results of filtering with IMPUTE info.

**Fig. 3.**
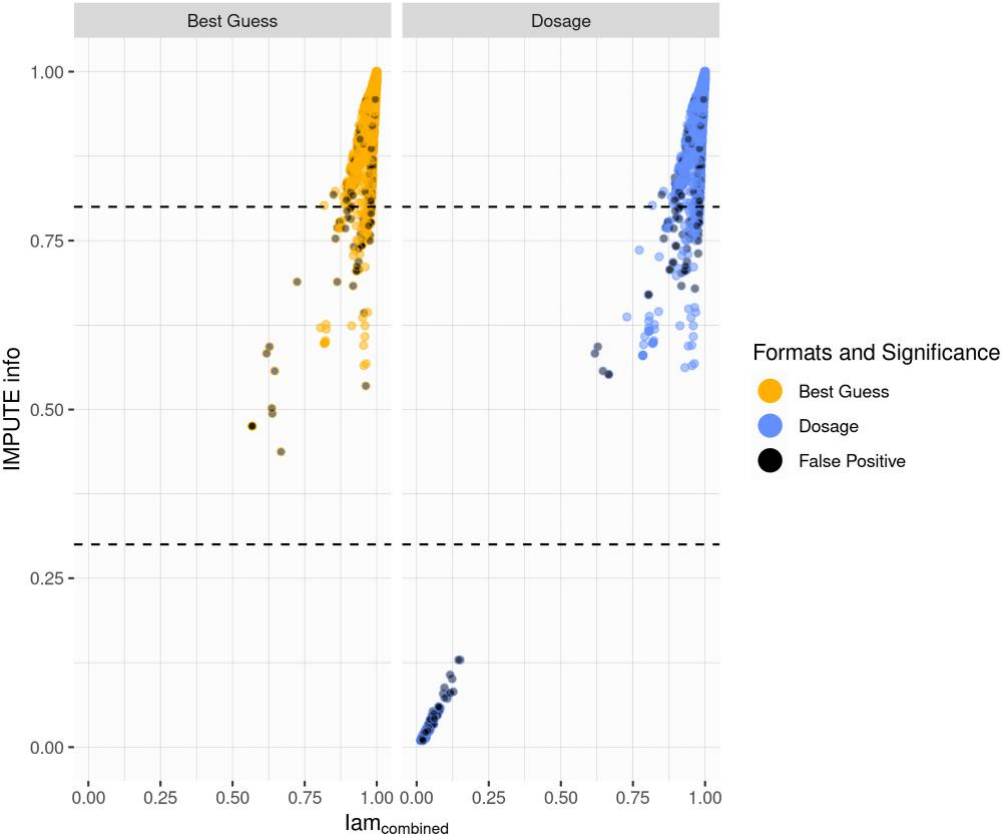
Scatterplot of significant and imputed SNPs with the IMPUTE info score on the *y*-axis and Iam_combined_ on the *x*-axis. The dashed lines represent the thresholds of 0.3 and 0.8 for IMPUTE info, indicating the midrange. The formats and significance are indicated by the color. If the association is false, the dot is filled in with black.

Compared with the lower IMPUTE info threshold, 14 additional SNPs are discarded with the individual Iam scores, 7 of which SNPs are true positives. Iam_combined_ scores similarly when compared with the established quality measures regarding lower quality SNPs, but diverts afterwards, where it tends to score higher, as depicted in Fig. 3. The midrange of Iam_combined_ itself contains only 22 SNPs, 10 of which are true positive. SNPs scoring relatively low in both IMPUTE info and Iam_combined_ are false positives. There is a slight gap visible in Fig. 3 between the false-positive SNPs with an IMPUTE info score of around 0.5 and the true SNPs, which does not occur in singular use of the either measure. Setting a threshold for Iam_combined_ at 0.7 discards 11 additional false-positive SNPs compared with using IMPUTE info on its own with 0.3, while setting it to 0.8 raises the number of additional discarded SNPs to a total of 22, including 10 true positive associations.

The suggested threshold of 0.9 in hiQ does not discard any SNPs. Discarding SNPs with hiQ below 0.95 results in 27 false-positive SNPs discarded. Of these false-positive SNPs, 5 would be wrongfully kept with the lower IMPUTE info threshold and one even with the additional use of Iam_combined_.

**Fig. 4.**
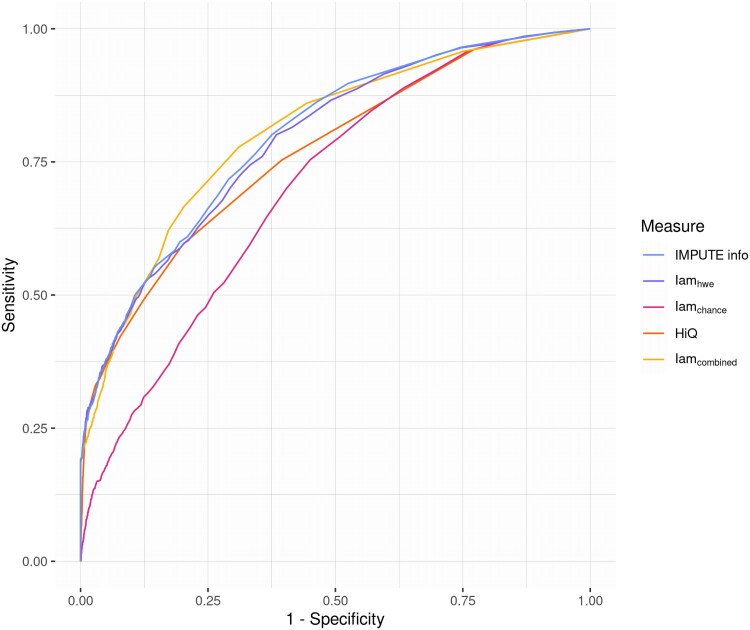
ROC curves of imputation quality measures. Sensitivity here is defined as correctly identifying false associations and specificity as correctly identifying true associations.

Figure 4 displays the receiver operating characteristics (ROC) curves for IMPUTE info, Iam_hwe_, Iam_chance_, HiQ, and Iam_combined_. In this setting, sensitivity is defined as the percentage of correctly discarded false associations and specificity as the percentage of correctly kept true associations. Iam_combined_ [area under the curve (AUC) = 0.803] is slightly better at classifying SNPs than IMPUTE info (AUC = 0.798). The ROC curve alone is not very informative for threshold setting, since there are more true SNPs than false ones. This puts the threshold for the separation with the highest sum of sensitivity and specificity between 0.988 and 0.997 for all measures. Fixing the specificity to a high value, so that the loss of true associations is limited, yields thresholds that are more reasonable and in line with practical use, which is displayed in Table 2. For those thresholds, the detection of false-positive SNPs is poor, further underlining the challenge of discerning between true and false associations.

**Table 2:** Thresholds for imputation quality measures for set values of specificity and AUC.

		Specificity > 0.99		Specificity = 1	
Measure	AUC	Threshold	Sensitivity	Threshold	Sensitivity
Impute info	0.798	0.83	0.26	0.56	0.19
Iam hwe	0.792	0.77	0.26	0.41	0.19
Iam chance	0.702	0.80	0.07	0.43	>0.01
HiQ	0.766	0.98	0.24	0.95	0.02
Iam combined	0.803	0.93	0.22	0.724	0.19

As in Fig. 4, sensitivity represents the proportion of false associations detected as such, as specificity represents the proportion of correctly identified true associations.

In general, the differences between using Iam and hiQ in addition to the lower IMPUTE info score are somewhat small, although additional false-positive SNPs may be discarded without sacrificing true positive SNPs.

MagicalRsq did not improve filtering on SNP basis. The calibration lowered the estimated imputation quality for most SNPs. The adjustment downward was more pronounced, if the population-specific allele frequencies were not included. This downward adjustment is then reflected in the results of filtering with the thresholds of 0.3 and 0.8. For some models, even a low threshold discards more than 50% of all SNPs for both formats. Notably, some models calibrated the imputation quality of the false-positive dosage-specific significant SNPs with very low imputation quality upwards. Compared with the unadjusted imputation quality measures, the number of SNPs passing either threshold are reduced, but the separation of false positive and true positive is not improved. Further details are given in Supplementary Table 2 and Fig. 2.

For both Iam hiQ and MagicalRsq, the numbers of false positives and true positives discarded are not much different from setting a different IMPUTE info threshold. The problem of the midrange of imputation quality remains: the majority of false-positive and true-positive SNPs are not separable.

### Filtering with imputation quality on aggregated SNPs

Including format-specific association signals, there are a total of 660 spikes. Spikes are considered true positive, if one or more true significant SNPs are present. Significant spikes containing both false-positive and false-negative SNPs are considered true associations. Filtering with singular imputation quality thresholds for spikes results in full deletion, partial, or no deletion. Partial spikes remain detectable and therefore should be regarded as still present after filtering, but might suffer a loss of confidence in interpretation.

Imputed significant SNPs within a spike containing significant typed SNPs generally have a very high imputation quality with 99.21% above 0.8 IMPUTE info and would not be discarded by any of the discussed methods. Therefore, the analysis focuses on the 304 spikes containing exclusively imputed SNPs, 180 of which are false. Separated by format, 15.87 and 58.42% of spikes detected with best guess and dosage, respectively, are false.

Table 3 presents the results of filtering aggregated SNPs separately for best guess and dosage, comparing thresholds for IMPUTE info and the Midrange Filter. There are 116 spikes containing only high-quality SNPs, which are kept by all methods, and 157 spikes containing only low-quality SNPs, which are discarded by all methods. The low-quality spikes are dosage specific. The remaining 31 spikes contain at least 1 SNP in the midrange, i.e. spikes for which the decision to discard differs between the 0.3 and 0.8 thresholds. Comparing the higher and lower IMPUTE info threshold for discarding SNPs and the resulting deletion of spikes, the lower threshold does not discard any true positive spikes. In turn, the lower threshold is not as effective at deleting false-positive spikes. The majority of spikes discarded by the higher threshold compared with the lower are true positive signals in both formats. The number of wrongfully discarded spikes in the midrange is higher for dosage, notably because almost every spike in the midrange is a true positive spike. In total, there are 124 true spikes, 18 of which are specific to dosage and 3 of which are specific to best guess. The simulation settings do not seem indicative on whether one format is able to identify the signal and the other is not, but in general, dosage identifies more significant and supporting SNPs than best guess, when both are able to detect the signal.

**Table 3.** Results of filtering with IMPUTE info in spikes.

	Best guess spikes			Dosage spikes		
	Total	False	True	Total	False	True
Imputed + typed	478	20	458	645	170	475
Imputed	126	20	106	291	170	121
>0.8 IMPUTE info	104 (+5)	12	92 (+5)	109 (+7)	10	99 (+7)
>0.3 IMPUTE info	126	20	106	131(+1)	10 (+1)	121
Midrange Filter	117	12	105	131	10	121

False+ and True+ are the number of false- and true-positive associations, respectively. The row “All” represents the unfiltered number of spikes, while “Imputed” counts spikes containing only imputed SNPs. Further filtering was applied on only the imputed spikes. The number of partially discarded but still present spikes are added in parentheses. Note that the numbers in the table do not add up intuitively, owing to partial deletions and format-specific associations. Results based on Beagle R² and MaCH R² are included in Supplementary Table 3.

**Fig. 5.**
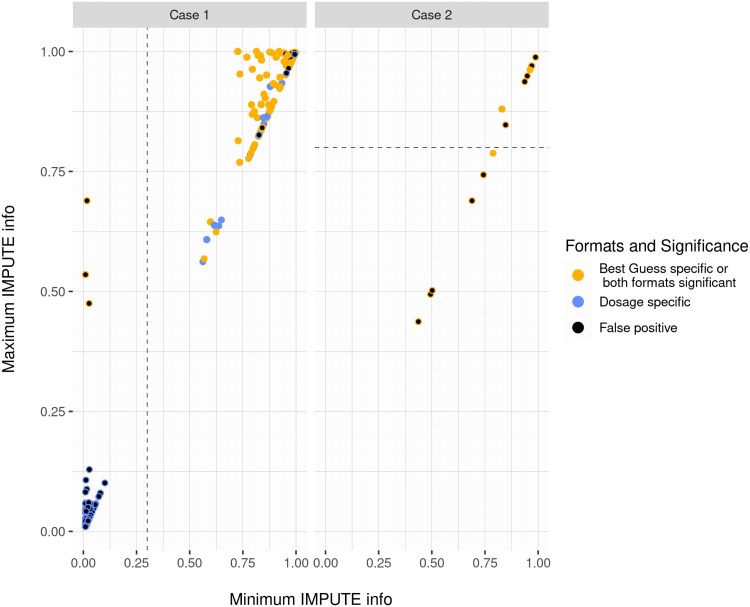
Midrange Filter decisions. The *x*-axis depicts the smallest and the *y*-axis the largest IMPUTE info score within each spike. The plot is split according to the type of spike relevant to the application of the Midrange Filter: specific to best guess on the left (Case 1) and dosage specific or both significant on the right (Case 2). The colors in the right plot indicate whether the spike is dosage specific. If the spike is false positive, the dot is filled in with black. The Midrange Filter discards spikes below the dashed lines for each type of spike.

Applying the Midrange Filter to the imputed spikes results in only one discarded true signal for any of the established imputation quality measures, but reduces the number of false-positive spikes remaining in the dataset to the level of discarding SNPs with a high imputation quality threshold. Figure 5 illustrates the classification of the spikes by the Midrange Filter into true- and false-positive signals. The Midrange Filter correctly identifies all spikes in the midrange as false or true positive except 1 spike, which stems from a simulation setting with a mismatched reference panel. Thus, our method outperforms using any singular threshold in discerning true- and false-positive association signals. Out of 180 false-positive spikes, 165 are discarded by the Midrange Filter based on IMPUTE info thresholds. The resulting data in best guess format contain 7 format-specific spikes, 5 of which are false positive. For dosage, 21 format-specific spikes remain in the dataset, 3 of which are false positive. Note that all SNPs of the remaining false-positive results pass the upper imputation quality threshold and would not be discarded by the individual threshold. Results for Beagle R² and MaCH R² are almost identical. Beagle R² scores slightly lower in some SNPs, which results in a few more discarded signals. The results are included in Supplementary Table 3 for the sake of completeness.

The presented results are based on the minimum imputation quality between cases and controls, since low imputation quality in even 1 group reduces confidence in the following association result. If the mean or maximum between cases and controls is taken, the Midrange Filter still performs well. The number of remaining false-positive spikes is comparable or the same as using the higher threshold, except in the dosage format using the maximum between cases and controls. In this specific case, the number of remaining false-positive spikes is halfway between results for the lower and upper threshold, while still preserving the majority of true signals as before. See Supplementary Table 4 for more detailed results. Note that the gap between values in the lower midrange displayed in Fig. 3 narrows or closes when using the mean or the maximum, respectively.

### Application of Midrange Filter—PsyCourse study

The GWAS did not result in any genome-wide significant association. There were 28 SNPs with a *P*-value of exactly 0, which we investigated and discarded due to lack of confidence in any significant findings. Table 4 presents the 80 SNPs found with a *P*-value below 5e−6 in either the best guess or dosage format, including the imputation quality directly taken from the imputed SNP file and the minimum between the recalculated imputation quality of the diagnostic groups for each SNP. The SNPs were grouped into 17 spikes, 6 of which consist of several SNPs with a strong signal. The majority of SNPs have an imputation quality over 0.8. There are 3 spikes specific to best guess and 4 specific to dosage. Two spikes contain genotyped SNPs and all imputed SNPs within these particular spikes are over 0.95 imputation quality. There were no SNPs found with an initial imputation quality lower than 0.3 in the surrounding SNPs with a comparably low *P*-value. However, recalculating the imputation quality separately for the diagnostic groups reveals 5 SNPs, which initially passed the 0.8 or the 0.3 threshold but fell below the threshold for the recalculated imputation quality. In total, the 0.8 threshold on the initial imputation quality discards 3 SNPs on initial imputation quality and 8 on the recalculated value. The SNP rs181612397 on chromosome 6 fell from an initial R² of 0.66 to a minimum imputation quality of 0.38 for IMPUTE info, 0.37 for Iam_hwe_, 0.24 for Beagle R². Depending on the imputation quality measure used, this SNP is discarded by either both thresholds and the Midrange Filter or only by the 0.8 threshold. Further, the Midrange Filter discards the SNP rs148599288 on chromosome 1 after recalculating the Imputation quality for diagnosis groups, which is a best guess specific signal. In contrast to the 0.8 threshold, the Midrange Filter preserves an association signal on chromosome 20 consisting of 2 SNPs. The SNP with the lowest *P*-value at 1.34e−7 is found on chromosome 2. There are 13 SNPs in close proximity with a *P*-value below 5e−6, all of which have an imputation quality score above 0.8 both initially and recalculated for diagnosis groups.

**Table 4:** SNPs below a *P*-value below 5e−6 of the GWAS on the PsyCourse data.

Location	*P*-value times e−6		Imputation quality		Location	*P*-value times e−6		Imputation quality	
	Dosage	BG	Initial	Min		Dosage	BG	Initial	Min
1:205680881	2.28	4.04	0.935	0.895	5:124428561	1.62	8.65	0.885	0.849
1:205717676	2.18	1.41	0.966	0.954	6: 43842319	1.30	3.68	0.817	0.704
1:205719191	1.85	1.07	0.971	0.966	6:130733300	4.65	>100	0.659	0.238
1:205719513	2.84	1.81	0.948	0.913	10:28800202	3.00	2.70	0.959	0.957
1:205728574	2.27	2.61	0.969	0.965	10:28801273	3.01	2.70	0.959	0.957
1:205735864	2.32	2.28	0.968	0.964	10:28805472	3.40	3.40	Typed	Typed
1:205737511	2.40	2.61	0.968	0.965	10:28821444	2.33	2.27	0.953	0.95
1:210978952	12.44	4.73	0.844	0.603	10:28830108	3.23	2.80	0.953	0.949
2:97014837	2.97	1.53	0.971	0.956	10:28832820	3.23	2.80	0.953	0.949
2:102161388	1.22	1.22	0.947	0.911	10:28843854	3.23	2.80	0.953	0.949
2:102162574	2.85	1.60	0.945	0.925	10:28862152	3.02	3.12	0.952	0.946
2:102170710	1.68	0.69	0.943	0.920	10:28869729	3.61	3.82	0.953	0.947
2:102172703	1.89	1.49	0.939	0.910	10:28880160	2.93	3.12	0.952	0.946
2:102173795	1.89	1.49	0.939	0.910	10:28880992	2.93	3.12	0.952	0.946
2:102174383	1.89	1.49	0.939	0.910	10:28886537	2.69	2.63	0.956	0.950
2:102175042	1.89	1.49	0.939	0.910	10:28886626	2.69	2.63	0.956	0.950
2:102175179	1.89	1.49	0.939	0.910	10:28891174	2.69	2.63	0.956	0.950
2:102177233	0.134	0.96	0.832	0.772	10:28893554	2.67	2.63	0.956	0.950
2:102177394	1.83	1.66	0.936	0.901	10:28894110	3.24	3.31	0.956	0.950
2:102177774	1.82	1.66	0.936	0.901	10:28894247	2.67	2.63	0.956	0.950
2:102177908	1.82	1.66	0.936	0.901	10:28905079	2.62	2.63	0.956	0.949
2:102177980	1.82	1.66	0.936	0.901	10:28913309	4.10	4.10	Typed	Typed
2:102178199	1.84	1.66	0.936	0.901	12:116890740	4.99	4.25	0.838	0.554
2:204723052	4.86	12.37	0.952	0.924	13:40886155	0.433	7.51	0.695	0.546
2:240877081	5.73	4.14	0.915	0.897	14:80936831	3.95	4.82	0.952	0.947
4:141091361	4.75	4.75	Typed	Typed	14:80947552	4.50	5.60	0.954	0.948
4:141104884	3.78	5.84	0.962	0.952	14:80947648	3.69	4.73	0.958	0.941
4:141110792	4.15	4.75	0.969	0.960	14:80947818	3.68	4.73	0.95	0.941
4:141111464	3.56	4.75	0.965	0.956	14:80948392	3.64	4.73	0.95	0.941
4:141117653	4.13	4.75	0.968	0.960	14:80949372	3.62	4.58	0.95	0.940
4:141120663	3.77	2.19	0.969	0.963	17:80667812	7.34	2.63	0.958	0.900
4:141121085	3.77	2.19	0.969	0.963	17:80668325	5.33	2.27	0.960	0.901
4:141125645	3.78	2.19	0.969	0.962	17:80668375	5.42	2.27	0.961	0.901
4:141128772	3.82	2.19	0.969	0.962	17:80668985	5.40	2.27	0.961	0.901
4:141132155	3.77	2.19	0.969	0.963	17:80669238	5.31	2.27	0.960	0.901
4:141136738	3.77	2.19	0.955	0.916	17:80670820	5.31	2.27	0.960	0.901
4:141138951	5.48	3.79	0.954	0.916	17:80670873	5.32	2.27	0.960	0.901
4:141141924	2.54	1.47	0.954	0.915	17:80671047	5.32	2.27	0.960	0.901
4:141150956	2.23	1.47	0.956	0.923	20:18926221	1.74	0.78	0.642	0.518
4:141152718	5.87	4.72	0.953	0.921	20:18954449	5.17	1.51	0.642	0.514

For each SNP, the *P*-value for both the dosage and the BG formats are listed. The column Imputation Quality “Initial” depicts the imputation quality taken directly from the file after imputation. Genotyped SNPs, where this does not apply, are labeled as such. The column Imputation Quality “Min” depicts the minimum of the recalculated imputation quality for diagnosis groups (Beagle R²).

BG, best guess.

## Discussion

In this work, we investigated the divergence of *P*-values from association analysis between gold standard and imputed SNPs, the rate of false-positive significance, and the impact of postimputation quality control. We illustrated the difficulties of filtering SNPs in the midrange of imputation quality and proposed a new method, the Midrange Filter, which filters association signals for false-positive results not on single SNPs but on spikes. In our results, the vast majority of spikes in the midrange are correctly identified as true- or false-positive signals.

The Midrange Filter is a promising method to preserve more true-positive associations without sacrificing imputation quality. Given that this work simulates smaller study settings, further research is needed both for larger sample sizes and testing in more real-data examples. The only true spike, which was discarded by the Midrange Filter, stems from a simulation setting with a mismatched reference panel. In this specific simulated dataset, the signal does appear at the specified location of the disease locus as set in the simulation, but the spike contains only one highly significant SNP with no close SNPs supporting the association with comparably low *P*-values. This signal would likely not be considered a high-confidence association when viewed in a Manhattan plot. The decision of the Midrange Filter in this particular case does not discard an unquestionably true signal.

Aggregating SNPs to spikes revealed that the majority of SNPs in the midrange in the dosage format are part of or entirely true signals. Using a high-quality threshold such as 0.8 for IMPUTE info is therefore more detrimental than expected and should be used with caution. The results of this work support the use of a lower threshold for dosage to discard false-positive SNPs, if a single threshold is to be used in the first place. In contrast, a low threshold for associations based on best guess SNPs does not discard any significant SNPs in this work, while the upper threshold discards both false- and true-positive signals. Therefore, neither threshold is superior to the other. This makes the Midrange Filter more effective for the best guess genotype than the dosage. Still, best guess finds fewer true format-specific spikes than dosage, as is expected given the power difference. Therefore, dosage is preferable as a basis for association in general, but the Midrange Filter performs well for both genotype formats.

The minimum imputation quality between separately imputed cases and controls is a very informative characteristic for dosage and a strong indicator of wrongful association, even if the imputation quality of the other group is high. This characteristic is not available to all GWAS settings, e.g. if the phenotype is continuous. However, the Midrange Filter performs well even if the mean or the maximum of imputation quality is taken, which might approximate such circumstances. In these cases, the Midrange Filter is more effective than the low threshold for both best guess and dosage. While this work is limited to the case–control setting, the Midrange Filter shows potential to wider applicability.

Given the low rates of false-positive spikes in both formats, discarding even more false-positive spikes without compromising on keeping true-positive associations is likely not possible with a postimputation method alone. The remaining false-positive spikes are about the same number as the undetected spikes, which reflects the both-sided divergence of *P*-values after imputation and the limits of postimputation quality control. As other research indicates, the fit and size of the reference panel has influence on the imputation accuracy (Das *et al*. 2016; Bai *et al*. 2019), as does the density of the SNP array (Nelson *et al*. 2013; Ha *et al*. 2014), even though we did not find a difference in our simulations for the latter. The effectiveness of the Midrange Filter depends on the presence of SNPs in the midrange of imputation quality. An increase in imputation quality might render this method less effective in the future, although there is a mathematical and biological limit to imputation quality, e.g. spontaneous mutations and rare variants. The majority of false spikes in the simulations appeared in settings with a small reference panel. We included simulation settings like these to increase the chance of producing false spikes without an excessive amount of iterations. This should not be taken as an assurance that false spikes cannot occur in GWAS with a sufficiently large reference panel.

The results gained with Iam_combined_ are comparable with more established imputation quality measures. In combination with, e.g. the IMPUTE info score as depicted in Fig. 3, it identifies false-positive–associated SNPs in the lower tail end of the midrange better. This translates to using Iam_combined_ in addition with a threshold of 0.7. An SNP indicated by both Iam and IMPUTE info is more likely to be a false-positive result instead of SNPs indicated by only one measure. Given the novelty of Iam, the placement of thresholds in Iam is still somewhat empirical (Rosenberger *et al*. 2022). The recommended threshold of hiQ was not informative in our simulation, most likely given our small number of participants in each dataset, which limits heterogeneity by default. Raising the threshold to 0.95 yielded small improvements. However, this was only possible to discover because the associations were known to be true or false positive, since there is no clear cluster of false-positive SNPs for hiQ. Given the AUC and the thresholds in Table 4, the recommended thresholds for Iam at 0.5 appear to be a balanced recommendation.

The models used in the calibration of MagicalRsq are meant to be trained on the actual study population for effective use, e.g. if a subset of participants is fully sequenced (Sun, Yang, *et al.* 2022). For small studies as the ones simulated in this work, this is often not the case. While this might be noteworthy for future studies, where training specific models is not possible, it is not indicative of to the actual effectiveness of MagicalRsq with a properly trained model. Since the size of datasets influences the outcome of imputation, it is possible that the calibration would be accurate, if imputation results similar to this work were produced by larger datasets.

The dosage format identifies a larger number of significant SNPs, both false positive and true positive. The majority of false-positive SNPs stemming from dosage score high in Iam_chance_, but low in Iam_hwe_, as can be derived from Fig. 3. Owing to the intuitive interpretation of Iam, it is very likely that these SNPs were only imputed according to allele frequencies without any individual information (Rosenberger *et al*. 2022). These SNPs are identified easily by all imputation quality measures. Therefore, dosage is the more favorable format to catch association signals because of its larger number of format-specific true-positive signals. Given that spikes specific to best guess are more likely to be false positive than true positive even with high imputation quality, they should be introduced with caution if the initial association is run on dosage.

The Midrange Filter has a limitation similar to the individual thresholds: the exact limit to the midrange of imputation quality is empirical. Setting the thresholds too high might result in discarded true positive spikes, while setting them too low might result in more remaining false-positive spikes. The thresholds 0.3 and 0.8 are well-established and therefore sensible benchmarks, but they are likely not optimal for every dataset. The Midrange Filter might profit from different thresholds especially for data imputed with IMPUTE2, since its imputation quality and the resulting quality measures differ from more recent imputation tools (Stahl *et al*. 2021), such as IMPUTE4, minimac4, and Beagle5.2, the last of which was used in this work.

However, one of the advantages of the Midrange Filter is the awareness of the spikes it discards within the midrange and the reason for the decisions. Therefore, researchers may and should still discuss spikes which the Midrange Filter discards. Further, we recommend examining spikes where the decision of the Midrange Filter seems inadequate. This might occur in general in regions, where the imputation or analysis is known to be difficult, such as the major histocompatibility complex (MHC) or chromosomes X and Y. It is possible that the Midrange Filter might discard a prominent spike with generally high imputation quality because of one badly imputed SNP on the very edge of the considered SNPs surrounding the spike. Otherwise, a directly genotyped but dubious SNP, i.e. a singular significant SNP without a spike, might gain supporting SNPs by imputation, in which case, the Midrange Filter would keep this spike. Even though the signal itself would not be completely induced by imputation, the imputed SNPs might lend it undue credibility. Note, that there were no spikes and decisions fitting either description in our simulations.

Another genotyping method relying on imputation, and therefore, imputation quality control is low-pass sequencing. Quality control for these datasets usually includes discarding imputed genotypes with a genotype probability below a certain threshold and not necessarily whole SNPs with a low imputation quality. Given that imputation quality measures are calculated with genotype probabilities, there seems to be a possibility to adapt the Midrange Filter to this setting, but without further research in this particular area we cannot propose specifics for such an adaptation and its efficacy. We expect effective threshold setting would need to be revised at the least. After genotypes below a certain genotype probability is discarded as is common praxis, imputation quality measures could be recalculated, and the comparison between those and the initial imputation quality might be informative about confidence in association results. This could also include an adjustment that punishes for the number of deleted genotypes within each SNP.

The sample sizes of the datasets are reflective of smaller studies such as clinical trials. Even though genotyping is more accessible, resulting in, e.g. studies with several hundreds of thousand participants and large population-based reference panels, studies with a similar or even smaller sample size are still conducted. The GWAS Catalog (Llinàs-Reglà *et al*. 2017) (www.ebi.ac.uk/gwas) lists 203 publications with <10,000 participants with a median of 1745 participants as recent as 2023 (Sollis *et al*. 2023; Cerezo *et al*. 2025). Owing to the small number of participants in each simulation, there is a mathematical limitation to MAF and therefore significance. However, the SNPs in the midrange are by a vast majority common SNPs. In the midrange defined by IMPUTE info, only 7.4% are SNPs with an MAF lower than 0.05; for the true-positive SNPs in the midrange, it is 1.2%. Therefore, the Midrange Filter does not target SNPs that are inherently harder to impute but provides new insights in imputation quality, which reflect the biological dependencies better than filtering on individual SNPs.

The application of the Midrange Filter to the PsyCourse study cannot be truly verified, since whole-genome sequencing data do currently not exist. The GWAS did not yield genome-wide significant results. Previous research performing GWAS for the mental diseases in this dataset suggest a lack of power due to a smaller sample size (Mallard *et al*. 2022; Trubetskoy *et al*. 2022; Meng *et al*. 2024; O’Connell *et al*. 2025). Spikes may have been found consisting of SNPs below the 0.3 imputation quality threshold, but those would be discarded for any quality control method and are therefore not of interest for this application. The spike containing the lowest *P*-value is close to the protein-coding gene interleukin 1 receptor type 2. This receptor is involved in the regulation of stress responses and has been proposed as a treatment target for depression (Koo and Duman 2009), which makes it a plausible association. The 2 SNPs discarded by the Midrange Filter are located within genes associated with phenotypes unrelated to the mental health diagnoses of the PsyCourse participants: KCNH1 is associated with epilepsy (Tian *et al*. 2023) and severe and rare development disorders (Gripp *et al*. 2021), while TMEM200A is associated with gastric cancer (Deng *et al*. 2023). No other SNPs were located within genes. SNPs in a spike with a significant typed SNP have consistently high imputation quality, which is consistent with our simulations and lends credibility to not filter spikes with significant typed SNPs. The recalculation of imputation quality separated by group reveals that one SNP in particular is not as well imputed as the initial R² implied. A joint imputation may mask poor imputation quality for one group, because the estimate is based on the overall performance. This is similar to imputation quality measures not reflecting missed rare alleles. Even though for the majority of SNPs with a low *P*-value, the difference between the initial and recalculated imputation quality is minimal, and recalculating imputation quality control for cases and controls separately may help indicate false associations.

## Supplementary Material

jkaf043_Supplementary_Data

## Data Availability

An implementation of the Midrange Filter and the code to generate the presented simulations and results are available at github.com/StahlKt/MidrangeFilter. The 1KGP are available through the International Genome Sample Resource at internationalgenome.org. The informed consent signed by participants of the PsyCourse Study does not allow their phenotypic and genomic data to be made publicly available. However, these data may be accessed upon reasonable request or research proposal via http://www.psycourse.de/proposals-en.php. We affirm that all data necessary for confirming the conclusions of this article are represented fully within the article and its tables and figures. Supplemental material available at G3 online.
