# Supplementary material for: Aggregating single nucleotide polymorphisms improves filtering for false-positive associations postimputation
Source: G3 (Bethesda). 2025 Mar 7;15(5):jkaf043. doi: 10.1093/g3journal/jkaf043 (PMC12060241; doi:10.1093/g3journal/jkaf043)
Supplement: jkaf043_Supplementary_Data [file jkaf043_supplementary_data.docx]

Supplement

Main text: Aggregating SNPs Improves Filtering for False Positive Associations Post-Imputation

Katharina Stahl^1^*, Sergi Papiol^2,3^, Monika Budde^2^, Maria Heilbronner^2^, Mojtaba Oraki Kohshour^2,4^, Peter Falkai^3,5,6^, Thomas G. Schulze^2,7,8,6^, Urs Heilbronner^2^, Heike Bickeböller^1^

*^1^Department of Genetic Epidemiology, University Medical Center Göttingen, 37073 Göttingen, Germany*

*^2^Institute of Psychiatric Phenomics and Genomics (IPPG), LMU University Hospital, LMU Munich, 80336 Munich, Germany*

*^3^Department of Psychiatry and Psychotherapy, LMU University Hospital, LMU Munich, 80336 Munich, Germany*

*^4^Department of Immunology, Faculty of Medicine, Ahvaz Jundishapur University of Medical Sciences, Ahvaz, Iran*

*^5^Max Planck Institute of Psychiatry, Department Clinical Translation, Munich, Germany*

*^6^German Center for Mental Health (DZPG), partner site Munich/Augsburg, Munich, Germany*

*^7^Department of Psychiatry and Behavioral Sciences, SUNY Upstate Medical University, Syracuse, NY, USA*

*^8^Department of Psychiatry and Behavioral Sciences, Johns Hopkins University School of Medicine, Baltimore, MD, USA*

*corresponding author

Content

[Random Deletion 2](#_Toc190205814)

[Results MagicalRsq 5](#_Toc190205815)

[Midrange Filter 7](#_Toc190205816)

[Simulation Details 8](#_Toc190205817)

[References 11](#_Toc190205818)

# Random Deletion

Extending the simulation settings from previous research (Zhang et al. 2022), we contrasted a random deletion pattern with SNP arrays as basis for imputation.

The results of the random deletion pattern were systematically different from those produced by simulating a SNP array. For comparison, simulating the SNP arrays corresponds to deleting 86%, 92% and 98% of SNPs contained in the data set in order of density.

In Table S1, the significant SNPs are grouped by genotype format and deletion pattern, as well as the number of false positive and inflated associations. A SNP and its association is considered false positive, if the level of significance is met only in the imputed data set, but not in the underlying simulation.

Random deletion causes a greater inflation and number of false positive SNPs. It is worth noting that the majority of significant SNPs discovered are false positive in the best guess format for random deletion, while in the SNP array simulations the dosage format introduces more false positives, albeit on a smaller scale. Figure S1 illustrates that the inflation for randomly deleted SNPs is also more severe. It follows that SNPs with a *P*-value close to *P* = 1 may test as highly significant after imputation. This is rarely and never the case for dosage and best guess, respectively, in the SNP array simulations we conducted.

Since the arrays contain even less SNPs, the amount of deleted SNPs is unlikely to cause this difference between deletion patterns. It might stem from randomly deleting highly informative SNPs that are included in the SNP arrays for this exact purpose. Also despite using a uniform distribution, the random deletion could lead to very sparse regions interfering with the imputation process. Low quality imputation could lead to stark differences between best guess and dosage as depicted in Figure 1 for several reasons. If SNPs are imputed just to reflect the MAF of the markers and the MAF differs between cases and controls, this would likely lead to significant findings in dosage but not best guess, similar to the easily identifiable false spikes we found in the array simulations. Additionally, if SNPs are imputed randomly, i.e. close to 1/3 for each genotype probability, dosage values would be close to 1 for all such genotypes, but best guess could fall on 0 or 2 prompted by slight variations such as (0.334, 0.333, 0. 333) or default to homozygous reference allele if probabilities are truly equal. This might produce large numbers of falsely associated SNPs specific to best guess.

Table S1: Overview of the number of significant SNPs of simulations with random deletion and deletion according to an existing SNP array. In the “Simulations” column, the number of simulations with significant associations are contrasted with the total number of simulations of the setting. False + depicts the false positive significant SNPs. Inflated indicates how many SNPs have a lower *P*-value after imputation than the true SNP genotype.

|  |  | Best Guess |  |  | Dosage |  |  |
| --- | --- | --- | --- | --- | --- | --- | --- |
|  | Simulations | Significant | False + | Inflated | Significant | False + | Inflated |
| Random | 178/234 | 107429 | 106837 | 107132 | 8590 | 7985 | 8328 |
| Array | 441/1032 | 11238 | 671 | 5509 | 11727 | 1012 | 6744 |


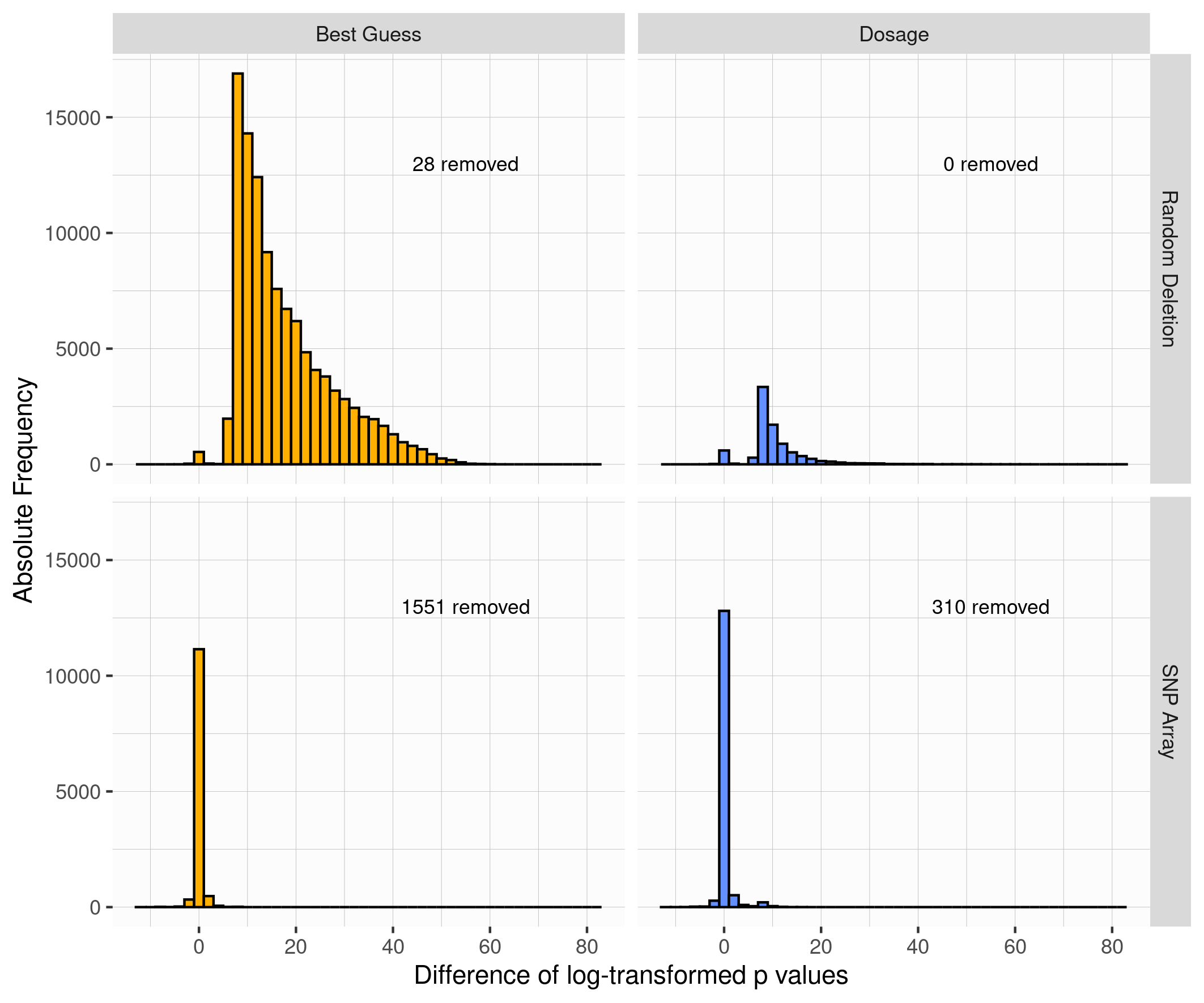


Figure S1: Histogram of differences between imputed and true *P*-values of significant SNPs, grouped by genotype format and deletion pattern. The *P*-values were log-transformed before subtracting the imputed value from the true value. Positive values on the x-axis indicate an inflation of *P*-values through imputation. For clarity, SNPs with a difference of 0 have been removed. The number of removed SNPs is indicated in the plot.

Figure S1 ALT TEXT: two times two plot of histograms, where the histograms with randomly deleted SNPs indicate large deviations in *P*-values, very pronounced in best guess. Only 28 and 0 SNPs for best guess and dosage respectively were identical between simulation and imputation in the random deletion scenarios, while in the imputation based on SNP arrays 1551 and 310 were identical. For SNP array based imputation, the majority of deviations is close to 0 on the logistical scale with a small number deviating on both sides.

# Results MagicalRsq


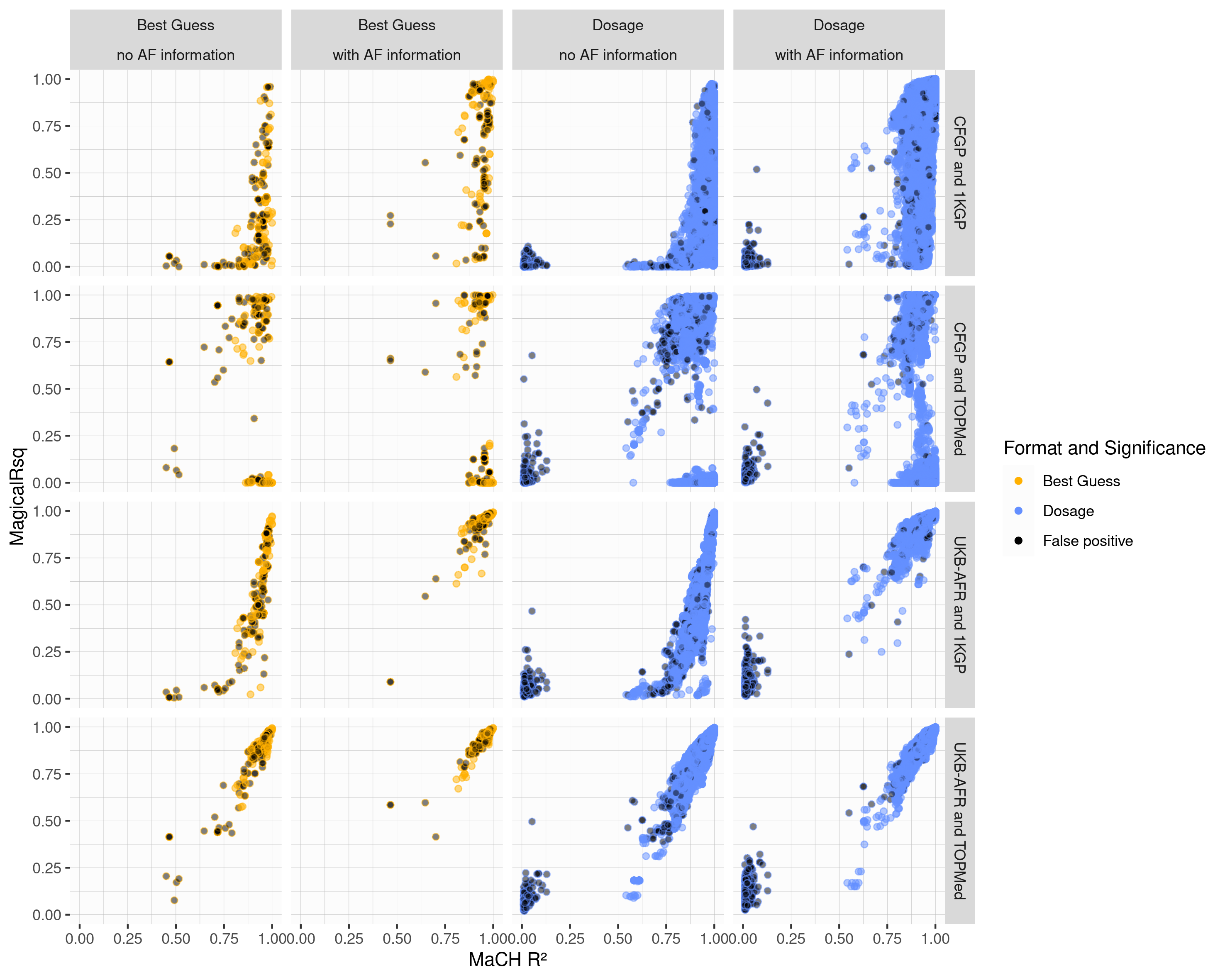


Figure S2: Scatterplot of MaCH R² and MagicalRsq for significant SNPs. Facets in one row are adjusted with the same model as labeled on the right side. The facet columns compare the effect of including population-specific allele frequencies (AF) for SNPs in each genotype format. The x-axis depicts the original MaCH R², the y- axis the adjusted MagicalRsq. If MagicalRsq adjusted the imputation quality down, the SNP is below the angle bisector.

Figure S2 ALT TEXT: Grid of scatterplots to illustrate the difference between initial MaCH R² and the recalibration with MagicalRsq. The grid has four columns and four rows. The first two columns depict significant SNPs in the best guess format and the last two columns depict significant SNPs in dosage format. For each format, the first column does not include information of population-specific allele frequencies, while the second does. The rows represent the models MagicalRsq trained on by the data sets involved in the following order: Cystic Fibrosis Genome Project imputed with 1000 Genomes Project, Cystic Fibrosis Genome Project imputed with TOPMed, UK Biobank with African descent imputed with 1000 Genomes Project, and UK Biobank with African descent imputed with TOPMed. In twelve plots, the recalibration lowers the imputation quality measures of high quality SNPs without being able to target false positive associations. In seven out of eight dosage plots, the quality of some of the false SNPs with low imputation quality are raised above 0.3 by MagicalRsq.

Table S2: Results for post-imputation quality filtering with MagicalRsq. The number of total, false positive (False+) and true positive (True+) SNPs are listed for each trained model and the results of filtering with a threshold of 0.8 and 0.3 based on MagicalRsq. The first half are the results without added information about population-specific allele frequencies (AF), the second half are the results with added AF. Compare to Table 2 in the main body of the paper.

|  | | Best Guess | | | Dosage | | |
| --- | --- | --- | --- | --- | --- | --- | --- |
| Model training data | threshold | Total | False+ | True+ | All | False+ | True+ |
| Magical Rsq without population specific allele frequencies added | | | | | | | |
| All SNPs | | 11238 | 671 | 10567 | 11713 | 998 | 10715 |
| CFGP \| 1KG | > 0.8 | 329 | 25 | 304 | 328 | 26 | 302 |
|  | > 0.3 | 4461 | 187 | 4274 | 4499 | 181 | 4318 |
| CFGP \| TOPMed | > 0.8 | 4060 | 322 | 3738 | 4169 | 342 | 3827 |
|  | > 0.3 | 4795 | 435 | 4360 | 4947 | 485 | 4462 |
| UKB-AFR \| 1KGP | > 0.8 | 3080 | 148 | 2932 | 3111 | 169 | 2942 |
|  | > 0.3 | 10115 | 532 | 9583 | 10237 | 554 | 9683 |
| UKB-AFR \| TOPMed | > 0.8 | 9311 | 465 | 8846 | 9419 | 493 | 8926 |
|  | > 0.3 | 11222 | 667 | 10555 | 11414 | 727 | 10687 |
| Magical Rsq with population specific allele frequencies added | | | | | | | |
| All | | 10510 | 506 | 10004 | 10889 | 775 | 10114 |
| CFGP \| 1KGP | > 0.8 | 5192 | 221 | 4971 | 5271 | 259 | 5012 |
|  | > 0.3 | 8809 | 388 | 8421 | 8926 | 414 | 8512 |
| CFGP \| TOPMed | > 0.8 | 3718 | 255 | 3463 | 3797 | 268 | 3529 |
|  | > 0.3 | 4380 | 295 | 4085 | 4482 | 318 | 4164 |
| UKB-AFR \| 1KGP | > 0.8 | 10254 | 485 | 9769 | 10373 | 510 | 9863 |
|  | > 0.3 | 10506 | 503 | 10003 | 10650 | 538 | 10112 |
| UKB-AFR \| TOPMed | > 0.8 | 9993 | 470 | 9523 | 10117 | 496 | 9621 |
|  | > 0.3 | 10504 | 506 | 9998 | 10641 | 537 | 10104 |

# Midrange Filter

Table S3: Complementary results of Table 3 in the main publication for post-imputation filter. The table displays the number of Spikes after using the listed post-imputation quality methods to filter SNPs in the formats best guess and dosage. The columns False + and True + display the number of false and true spikes respectively. The filtering methods are indicated by the imputation quality measures. The filtering methods are the singular thresholds 0.3 and 0.8, and the Midrange Filter (MRF). The number of partially deleted spikes are indicated in parenthesis.

|  | | Best Guess | | | Dosage | | |
| --- | --- | --- | --- | --- | --- | --- | --- |
| Quality control methods | | Total | False + | True + | Total | False + | True + |
| Beagle R² | > 0.8 | 86 (+14) | 11 | 75 (+14) | 89(+17) | 9 | 80(+17) |
|  | > 0.3 | 125 | 19 | 106 | 131 (+1) | 10 (+1) | 121 |
|  | MRF | 116 | 11 | 105 | 131 | 10 | 121 |
| MaCH R² | > 0.8 | 91 (+11) | 12 | 79 (+11) | 94(+14) | 10 | 84(+14) |
|  | > 0.3 | 126 | 20 | 106 | 131 (+1) | 10 (+1) | 121 |
|  | MRF | 117 | 12 | 105 | 131 | 10 | 121 |

Table S4: Results for post-imputation filter using the mean or the maximum of imputation quality measures between cases and controls to represent each SNP. As in Table S3, the number of significant spikes remaining after the indicated method are displayed. Partly discarded spikes are indicated in parenthesis. Compare to Table in the main publication and Table S3 above.

|  | | Best Guess | | | Dosage | | |
| --- | --- | --- | --- | --- | --- | --- | --- |
| Quality control methods | | Total | False + | True + | Total | False + | True + |
| Case Control Conciliation: Mean | | | | | | | |
| IMPUTE info | > 0.8 | 108 (+3) | 12 | 94 (+3) | 103(+5) | 10 | 103 (+5) |
|  | > 0.3 | 126 | 20 | 106 | 132 (+4) | 11 (+4) | 121 |
|  | MRF | 118 | 12 | 106 | 132 | 11 | 121 |
| Beagle | > 0.8 | 94(+11) | 12 | 82 (+11) | 97 (+13) | 9 | 88(+13) |
|  | > 0.3 | 126 | 20 | 106 | 131( +1) | 10 ( +1) | 121 |
|  | MRF | 117 | 12 | 105 | 131 | 10 | 121 |
| MACH | > 0.8 | 101 ( +8) | 12 | 89 (+8) | 106( +10) | 10 | 96 (+10) |
|  | > 0.3 | 126 | 20 | 106 | 132 (+4) | 11 (+4) | 121 |
|  | MRF | 117 | 12 | 105 | 132 | 11 | 121 |
| Case Control Conciliation: Maximum | | | | | | | |
| IMPUTE info | > 0.8 | 111 (+3) | 12 (+1) | 99 (+2) | 117 (+4) | 10(+1) | 107(+3) |
|  | > 0.3 | 126 | 20 | 106 | 150 (+20) | 29 (+20) | 121 |
|  | MRF | 118 | 12 | 106 | 150 | 29 | 121 |
| Beagle R² | > 0.8 | 102( +7) | 12 | 90 ( +7) | 105 (+12) | 10 (+1) | 95 (+11) |
|  | > 0.3 | 126 | 20 | 106 | 142 (+14) | 21 (+14) | 121 |
|  | MRF | 117 | 12 | 105 | 142 | 21 | 121 |
| MaCH R² | > 0.8 | 107 ( +5) | 912(+1) | 95 (+4) | 116 (+3) | 10(+1) | 106 (+4) |
|  | > 0.3 | 126 | 20 | 106 | 149 (+21) | 28 (+21) | 121 |
|  | MRF | 117 | 12 | 105 | 149 | 28 | 121 |

# Simulation Details

Table S5: Details of the simulation settings. The table lists the used sets of disease loci, the deletion Pattern and the number of participants in both test data and reference panels. The number of individuals in the target data set for imputation is separated by cases and controls in this sequence. The number of individuals in the reference panel is equal for both cases and controls and therefore only listed once. The “mismatched” label refers to simulations, where the subpopulations between the reference panel and the test data set do not overlap. See Table S4 and Table S5 for details on the disease loci sets.

| Disease Loci | Deletion Pattern | Individuals Imputation | Individuals Reference |
| --- | --- | --- | --- |
| Set 1 | Random | 1000 \| 1000 | 10.000 |
| Set 1 | Random | 333 \| 1000 | 10.000 |
| Set 1 | Illumina Omni 5 | 1000 \| 1000 | 5.000 |
| Set 1 | Illumina Omni 25 | 1000 \| 1000 | 10.000 |
| Set 1 | Illumina Omni 25 | 1000 \| 1000 | 5.000 |
| Set 1 | Illumina Omni Express | 1000 \| 1000 | 10.000 |
| Set 1 | Illumina Omni Express | 1000 \| 1000 | 5.000 |
| Set 1 | Illumina Omni Express | 333 \| 1000 | 10.000 |
| Set 1 | Illumina Omni 5 | 1000 \| 1000 | Mismatched 10.000 |
| Set 1 | Illumina Omni Express | 1000 \| 1000 | Mismatched 10.000 |
| Set 1 | Illumina Omni 5 | 333 \| 1000 | Mismatched 10.000 |
| Set 2 | Illumina Omni 5 | 1000 \| 1000 | 10.000 |
| Set 2 | Illumina Omni 5 | 333 \| 1000 | 10.000 |
| Set 2 | Illumina Omni 25 | 1000 \| 1000 | 10.000 |
| Set 2 | Illumina Omni 25 | 333 \| 1000 | 10.000 |
| Set 2 | Illumina Omni Express | 1000 \| 1000 | 10.000 |
| Set 2 | Illumina Omni Express | 333 \| 1000 | 10.000 |
| Set 2 | Illumina Omni 25 | 1000 \| 1000 | Mismatched 10.000 |
| Set 3 | Random | 1000 \| 1000 | 10.000 |
| Set 3 | Random | 1000 \| 1000 | 5.000 |
| Set 3 | Random | 333 \| 1000 | 10.000 |
| Set 3 | Illumina Omni 5 | 1000 \| 1000 | 10.000 |
| Set 3 | Illumina Omni 5 | 333 \| 1000 | 10.000 |
| Set 3 | Illumina Omni 25 | 1000 \| 1000 | 10.000 |
| Set 3 | Illumina Omni 25 | 333 \| 1000 | 10.000 |
| Set 3 | Illumina Omni express | 1000 \| 1000 | 10.000 |
| Set 3 | Illumina Omni 5 | 1000 \| 1000 | Mismatched 10.000 |
| Set 4 | Illumina Omni 5 | 1000 \| 1000 | 5.000 |
| Set 4 | Illumina Omni 5 | 1000 \| 1000 | 10.000 |
| Set 4 | Illumina Omni 5 | 333 \| 1000 | 10.000 |
| Set 4 | Illumina Omni 25 | 1000 \| 1000 | 10.000 |
| Set 4 | Illumina Omni 25 | 333 \| 1000 | 10.000 |
| Set 4 | Illumina Omni Express | 1000 \| 1000 | 10.000 |
| Set 4 | Illumina Omni 5 | 1000 \| 1000 | Mismatched 10.000 |

Table S6: Details on disease loci sets. Listed are the purpose of the sets in the first column, followed by the exact positions and the MAF taken from the 1000 Genomes Project data set. The fourth column lists the used effect sizes for the heterozygote genotype (het) and homozygote genotype for the alternate allele (hom alt). Each position was combined with each effect in sets 1, 2 and 3. For combinations of set 4, refer to Table S5.

| Set | Positions | MAF simulation basis | Effect (het \| hom alt) |
| --- | --- | --- | --- |
| Set 1 : 45 simulations  Spread Positions,  medium effect sizes,  one disease locus in each simulation,  one simulation with each position and each effect | 4944596  10517696  16511171  22397853  30569039  36854795  43386156  48959642  54106271 | 0.3055  0.3494  0.2240  0.2045  0.1965  0.2240  0.0986  0.1845  0.2424 | 1.5 \| 1.5  1 \| 2  2 \| 2  1.5\| 2  1.5 \| 3 |
| Set 2 : 42 simulations  Spread Positions,  medium effect sizes, expand Set 1  one disease locus in each simulation,  one simulation with each position and each effect | 8971185  17707048  23624199  29417130  36751245  40816962  53847868 | 0.3227  0.0703  0.1885  0.1250  0.3343  0.1306  0.1058 | 1.5\| 1.5  1 \| 2  2 \|2  1.5 \| 2  1.5 \| 3  2 \| 3 |
| Set 3: 48 simulations  Marginal Positions,  smaller effect sizes, hinders imputation,  one disease locus in each simulation,  one simulation with each position and each effect | 1109907  2369674  2572057  3510356  4744393  4851896 | 0.1573  0.2664  0.1166  0.3307  0.2288  0.3794 | 1 \| 1.2  1.2\| 1.2  1\| 1.5  1.5\| 1.5  1\| 2  2\| 2  1.5 \| 2  1.5 \| 3 |
| Set 4: 45 simulations  Spread positions,  Medium effect sizes,  Multiple disease loci in each simulation | Combinations of positions from  Set 1 and Set 2,  See Supplementary Table 5 for details |  | Combinations of effects from  Set 1 and Set 2,  See Supplementary Table 5 for details |

Table S7: Details on disease locus set 4. This set contains several disease loci in each simulation. Positions and corresponding effect sizes are listed in the same format as Table S4. If Position 3 and Effect 3 are empty, then this simulation only contained two disease loci.

| Position 1 | Effect 1 | Position 2 | Effect 2 | Position 3 | Effect 3 |
| --- | --- | --- | --- | --- | --- |
| 10517696 | 1 \| 2 | 17707048 | 1.5 \| 3 | 48799716 | 1.5 \| 2 |
| 10517696 | 1.5 \| 1.5 | 8971185 | 1 \| 2 | 48799716 | 1.5 \| 2 |
| 10517696 | 1.5 \| 2 | 36751245 | 1.5 \| 3 | 48799716 | 1.5 \| 2 |
| 10517696 | 1.5 \| 3 | 53847868 | 1 \| 2 | 48799716 | 1.5 \| 2 |
| 10517696 | 2 \| 2 | 29417130 | 1 \| 2 | 48799716 | 1.5 \| 2 |
| 16511171 | 1 \| 2 | 17707048 | 2 \| 3 | 48799716 | 1.5 \| 2 |
| 16511171 | 1.5 \| 1.5 | 8971185 | 2 \| 2 | 48799716 | 1.5 \| 2 |
| 16511171 | 1.5 \| 2 | 36751245 | 2 \| 3 | 48799716 | 1.5 \| 2 |
| 16511171 | 1.5 \| 3 | 53847868 | 2 \| 2 | 48799716 | 1.5 \| 2 |
| 16511171 | 2 \| 2 | 29417130 | 2 \| 2 | 48799716 | 1.5 \| 2 |
| 22397853 | 1 \| 2 | 23624199 | 1.5 \| 1.5 | 48799716 | 1.5 \| 2 |
| 22397853 | 1.5 \| 1.5 | 8971185 | 1.5 \| 2 | 48799716 | 1.5 \| 2 |
| 22397853 | 1.5 \| 2 | 40816962 | 1.5 \| 1.5 | 48799716 | 1.5 \| 2 |
| 22397853 | 1.5 \| 3 | 53847868 | 1.5 \| 2 | 48799716 | 1.5 \| 2 |
| 22397853 | 2 \| 2 | 29417130 | 1.5 \| 2 | 48799716 | 1.5 \| 2 |
| 30569039 | 1 \| 2 | 23624199 | 1 \| 2 | 48799716 | 1.5 \| 2 |
| 30569039 | 1.5 \| 1.5 | 8971185 | 1.5 \| 3 | 48799716 | 1.5 \| 2 |
| 30569039 | 1.5 \| 2 | 40816962 | 1 \| 2 | 48799716 | 1.5 \| 2 |
| 30569039 | 1.5 \| 3 | 53847868 | 1.5 \| 3 | 48799716 | 1.5 \| 2 |
| 30569039 | 2 \| 2 | 29417130 | 1.5 \| 3 | 48799716 | 1.5 \| 2 |
| 36854795 | 1 \| 2 | 23624199 | 2 \| 2 |  |  |
| 36854795 | 1.5 \| 1.5 | 8971185 | 2 \| 3 |  |  |
| 36854795 | 1.5 \| 2 | 40816962 | 2 \| 2 |  |  |
| 36854795 | 1.5 \| 3 | 53847868 | 2 \| 3 |  |  |
| 36854795 | 2 \| 2 | 29417130 | 2 \| 3 |  |  |
| 43386156 | 1 \| 2 | 23624199 | 1.5 \| 2 |  |  |
| 43386156 | 1.5 \| 1.5 | 17707048 | 1.5 \| 1.5 |  |  |
| 43386156 | 1.5 \| 2 | 40816962 | 1.5 \| 2 |  |  |
| 43386156 | 1.5 \| 3 | 8971185 | 1.5 \| 1.5 |  |  |
| 43386156 | 2 \| 2 | 36751245 | 1.5 \| 1.5 |  |  |
| 48959642 | 1 \| 2 | 23624199 | 1.5 \| 3 |  |  |
| 48959642 | 1.5 \| 1.5 | 17707048 | 1 \| 2 |  |  |
| 48959642 | 1.5 \| 2 | 40816962 | 1.5 \| 3 |  |  |
| 48959642 | 1.5 \| 3 | 8971185 | 1 \| 2 |  |  |
| 48959642 | 2 \| 2 | 36751245 | 1 \| 2 |  |  |
| 4944596 | 1 \| 2 | 17707048 | 1.5 \| 2 |  |  |
| 4944596 | 1.5 \| 1.5 | 8971185 | 1.5 \| 1.5 |  |  |
| 4944596 | 1.5 \| 2 | 36751245 | 1.5 \| 2 |  |  |
| 4944596 | 1.5 \| 3 | 53847868 | 1.5 \| 1.5 |  |  |
| 4944596 | 2 \| 2 | 29417130 | 1.5 \| 1.5 |  |  |
| 54106271 | 1 \| 2 | 23624199 | 2 \| 3 |  |  |
| 54106271 | 1.5 \| 1.5 | 17707048 | 2 \| 2 |  |  |
| 54106271 | 1.5 \| 2 | 40816962 | 2 \| 3 |  |  |
| 54106271 | 1.5 \| 3 | 8971185 | 2 \| 2 |  |  |
| 54106271 | 2 \| 2 | 36751245 | 2 \| 2 |  |  |

# References

Zhang Z, Xiao X, Zhou W, Zhu D, Amos CI. 2022. False positive findings during genome-wide association studies with imputation: influence of allele frequency and imputation accuracy. Hum Mol Genet. 31(1):146–155. doi:10.1093/hmg/ddab203. https://doi.org/10.1093/hmg/ddab203.
